# Platinum nanoparticles labelled with iodine-125 for combined “chemo-Auger electron” therapy of hepatocellular carcinoma

**DOI:** 10.1039/d3na00165b

**Published:** 2023-05-12

**Authors:** Kamil Wawrowicz, Kinga Żelechowska-Matysiak, Agnieszka Majkowska-Pilip, Mateusz Wierzbicki, Aleksander Bilewicz

**Affiliations:** a Centre of Radiochemistry and Nuclear Chemistry, Institute of Nuclear Chemistry and Technology Dorodna 16 St. 03-195 Warsaw Poland k.wawrowicz@ichtj.waw.pl; b Department of Nuclear Medicine, Central Clinical Hospital of the Ministry of the Interior and Administration Wołoska 137 St. 02-507 Warsaw Poland; c Department of Nanobiotechnology, Institute of Biology, Warsaw University of Life Sciences Ciszewskiego 8 St. 02-786 Warsaw Poland

## Abstract

Convenient therapeutic protocols against hepatocellular carcinoma (HCC) exhibit low treatment effectiveness, especially in the context of long-term effects, which is primarily related to late diagnosis and high tumor heterogeneity. Current trends in medicine concern combined therapy to achieve new powerful tools against the most aggressive diseases. When designing modern, multimodal therapeutics, it is necessary to look for alternative routes of specific drug delivery to the cell, its selective (with respect to the tumor) activity and multidirectional action, enhancing the therapeutic effect. Targeting the physiology of the tumor makes it possible to take advantage of certain characteristic properties of the tumor that differentiate it from other cells. In the present paper we designed for the first time iodine-125 labeled platinum nanoparticles for combined “chemo-Auger electron” therapy of hepatocellular carcinoma. High selectivity achieved by targeting the tumor microenvironment of these cells was associated with effective radionuclide desorption in the presence of H_2_O_2_. The therapeutic effect was found to be correlated with cell damage at various molecular levels including DNA DSBs and was observed in a dose-dependent manner. A three-dimensional tumor spheroid revealed successful radioconjugate anticancer activity with a significant treatment response. A possible concept for clinical application after prior *in vivo* trials may be achieved *via* transarterial injection of micrometer range lipiodol emulsions with encapsulated ^125^I-NP. Ethiodized oil gives several advantages especially for HCC treatment; thus bearing in mind a suitable particle size for embolization, the obtained results highlight the exciting prospects for the development of PtNP-based combined therapy.

## Abstract 2

Here we present for the first time iodine-125 labeled platinum nanoparticles targeting the tumor microenvironment for combined “chemo-Auger electron” therapy of hepatocellular carcinoma.

## Graphical abstract

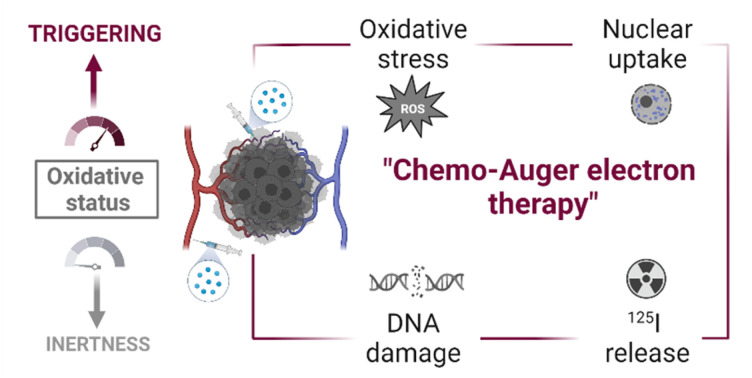

## Introduction

It is generally accepted that metastasis is one of the leading reasons for recurrence and the consequent cancer mortality.^1^ Dissemination of cancer sites consisting of single cells throughout the whole organism frequently located far away from the primary tumor site makes effective treatment by surgery or external radiotherapy impossible. Current therapeutic protocols include systemic chemotherapy as a major treatment option to deal with metastatic sites formed during cancer progression. The risk of severe side effects often makes it difficult or unreasonable to continue the treatment, especially when a high tumor burden on critical organs occurs. Therefore, there is an urgency to investigate effective therapies that can be primarily oriented towards micrometastases.

Targeted radionuclide therapy (TRT) based on short-range radiation-emitting radionuclides improves the treatment of such disseminated cancers by using radiolabeled compounds that specifically target tumor cells. These conditions are perfectly met by α and Auger emitters (AEs), high linear energy transfer (LET) radionuclides, which exhibit impressively precise and effective radio cytotoxicity, particularly by lethal DNA double-stranded breaks (DNA DSBs).^2^ The tissue range of α-emitters and AEs is limited to no more than several cells (α, 40–100 μm) or even restricted to single cells (AE, 1–20 nm). Unfortunately, despite achieving spectacular therapeutic effects,^3^ α-therapy cannot be used more widely due to the low availability of α-emitters. Current supplies of radioactive actinium (^225^Ac, ∼1.7 Ci per year), which is the most popular α-emitter in nuclear medicine, are sufficient only for preclinical studies and for the therapies of a few hundred patients worldwide. Recent intensive work on the cyclotron production of ^225^Ac in the spallation reaction on thorium (^232^Th(p,x)^227,225^Ac) and by the proton irradiation of radium (Ra; ^226^Ra(p,2n)^225^Ac) did not produce the expected results, mainly due to the contamination of ^225^Ac by the long half-life of ^227^Ac (*t*_1/2_ = 20 years).^4,5^

AEs may be a noteworthy alternative to achieve precise cell targeting in TRT. Improved availability and radiochemical properties can make AEs an excellent tool for enhancing the selectivity of treatment on the molecular level of an individual cell. The nuclear pathway of AEs involves decay *via* electron capture (EC) or internal conversion (IC) which leads to the formation of vacancies in the electronic shells. Rapid filling by electrons relaxing from external orbitals is accompanied by characteristic X-rays or cascades of Auger electrons. Moreover, these characteristic X-rays and Auger electrons offer an encouraging prospect for inducing a significant therapeutic response. Contrary to α-emitters, there is a wide range of Auger electron emitters that can be applied in TRT. The most commonly used AEs are radioactive indium (In; ^111^In), iodine (I; ^125^I), gallium (Ga; ^67^Ga), and technetium (Tc; ^99m^Tc). Because all of these radionuclides emit γ-quanta in high yield, they are used extensively in radiodiagnosis. They are also commonly available and well-characterized.

Auger electron emitters located in the DNA were found to be more radiotoxic than the α particle emitter polonium (Po; ^210^Po).^6^ Furthermore, in contrast to α and β^−^ radiation, AEs remain nontoxic while traveling in the blood or bone marrow. Conversely, AEs become highly efficient when located in the cell nucleus and incorporated into the DNA of the target cells. For these reasons, Auger radiotherapy is considered a promising emerging field in nuclear medicine, especially for targeting individual cancer cells and small tumor metastases as well as post-operative supportive therapy.^7,8^

The main restriction for the clinical application of AEs is the design of appropriate delivery systems which meet the rigorous criteria for the efficient and selective delivery of the high activities of Auger-based radiopharmaceuticals. Because most of the energy released by Auger electrons is deposited within proximity to the decay site, effective intracellular uptake is a minimum requirement, while targeting more sensitive organelles (*e.g.* mitochondria or the cell membrane^9^) is preferable. However, the most vulnerable target for an AE is DNA. Thus, nuclear uptake is another prerequisite for improving therapeutic efficacy. Apart from the direct interaction of Auger electrons with DNA, single- and double-stranded DNA breaks can also be indirectly induced by hydroxyl radicals (˙OH) and other reactive oxygen species (ROS) generated by Auger electrons in the cytosol. Therefore, multifunctionality is another advantage of this concept.

The therapeutic effect is strongly correlated with the high specific activity of a designed radiopharmaceutical. Direct attachment of radionuclides to biomolecules through chelators does not result in sufficient specific activities. For example, the specific activity of radiolabeled antibodies was relatively small (0.24 MBq μg^−1^), but several different strategies have been implemented to overcome this shortcoming.^10,11^ Moreover, many scientific reports have investigated the use of gold (Au) nanoparticles (NPs; AuNPs) as carriers of AEs.^12–16^

In our work, we propose to use platinum nanoparticles (PtNPs) and platinum coated gold core–shell nanoparticles (Au@Pt NPs) as transporters of Auger radiation emitters. Metastable Pt radionuclides (^193m^Pt and ^195m^Pt) are one of the most effective Auger electron emitters with great potency for medical applications. Unfortunately, it is almost impossible to obtain sufficient activities to carry out the therapy.^17,18^ According to the most promising procedure,^19^ we investigated the possibility of producing this nuclide indirectly, *via* neutron irradiation using an iridium target according to the two step reaction: 
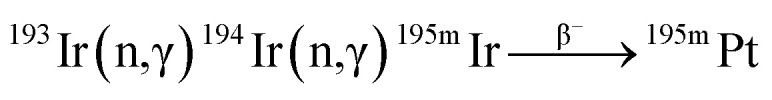
. Weekly irradiation of the ^193^Ir target in the Maria reactor in Świerk (Poland) with a 1.5 × 10^14^ n cm^2^ s^−1^ neutron flux yielded only ∼100 MBq for 1 mg of the target. Due to side reactions resulting in ^192^Ir and ^194^Ir production and difficulties in processing the target material, increasing the target mass and thus implementing this method was not reported.

**Fig. 1:**
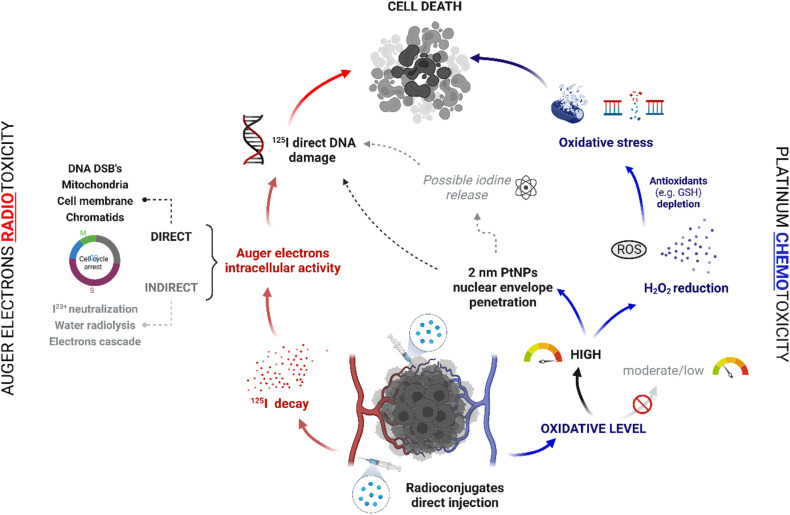
Postulated mechanism of combined “chemo-Auger electron” therapy.

Therefore, in our work, we did not pursue the application of ^195m^Pt. Instead, another effective AE, ^125^I, was utilized. For ^125^I decay associated with DNA, a “one decay = one DNA DSB” rule was postulated,^20,21^ which should correlate with high therapeutic effectiveness. To attach ^125^I to the surface of NPs we utilized the strong affinity of iodine atoms for adsorption on the surface of noble metals. We used ^125^I-coated PtNPs/Au@Pt NPs for hepatocellular carcinoma (HCC), the most common type of primary liver cancer in adults and currently the third leading cause of cancer-related deaths worldwide.^22^ We chose this type of cancer because HCC cells (HepG2) contain abnormally high levels of hydrogen peroxide (H_2_O_2_)^23,24^ and we hypothesized that in the presence of H_2_O_2_, ^125^I oxidation will occur on the surface of Au@Pt NP/PtNP, and ^125^I will easily release from the NP and freely diffuse near the DNA. Therefore, the proposed system should meet the criteria that are necessary for effective Auger electron therapy. In addition, in our previous work,^25^ we noted the high toxicity of nonradioactive Au@Pt NPs/PtNPs towards these cells related to the catalytic decomposition of H_2_O_2_ into more reactive ROS radicals on the NP surface and intranuclear uptake of PtNPs. Thus, such a system should enable selective chemo- and radiotoxic effects on HepG2 cells without a targeting moiety (Fig. 1).

## Experimental

### Reagents

Chemical reagents used: gold chloride(iii) trihydrate (HAuCl_4_·3H_2_O); sodium hexachloroplatinate hexahydrate (Na_2_PtCl_6_·6H_2_O); hexachloroplatinic acid (H_2_PtCl_6_); sodium rhodizonate (C_6_Na_2_O_6_); sodium hydroxide (NaOH); ascorbic acid (C_6_H_8_O_6_); thiolated carboxylic-polyethylene 4α; sulfanyl-ω carboxy PEG (HS-PEG-COOH, 5 kDa); hydrogen peroxide 30% (H_2_O_2_); bovine serum albumin (BSA); Triton X-100; methyl alcohol (MeOH); anti-phospho-Histone H2A.X (Ser139) antibody, clone JBW301; anti-mouse IgG (H + L), CF™ 633 antibody produced in goat; staurosporine; sodium carbonate and bicarbonate and TBS (tris buffered saline) were purchased from Merck & Co., Inc. (Kenilworth, NJ, USA). Hoechst 33 258 was purchased from Thermo Fischer Scientific (Waltham, MA, USA).

For biological experiments the following materials were used: EMEM; trypsin EDTA solution C; water, cell culture grade; phosphate-buffered saline (PBS), and fetal calf serum from Biological Industries (Beth Haemek, Israel). The CellTiter96® AQueous One Solution Reagent (MTS compound) from Promega (Mannheim, Germany). HepG2 cells were obtained from the American Type Tissue Culture Collection (ATCC, Rockville, MD, USA) and cultured according to the ATCC protocol. For experimental applications, over 80% confluent cells were used. All solutions were prepared with ultrapure deionized water (18.2 MΩ cm, Hydrolab, Straszyn, Poland).

### Radionuclides

^125^I (*t*_1/2_ = 59.5 days) and ^131^I (*t*_1/2_ = 8.01 days) were obtained from the National Centre for Nuclear Research, POLATOM Radioisotope Centre (Świerk, Poland). Specific activity was >600 GBq mg^−1^ and >550 GBq mg^−1^ for iodine-125 and iodine-131 respectively. Both radionuclides were supplied in aqueous solution of sodium iodine (NaI) with pH around 10–12 adjusted through sodium hydroxide or sodium carbonate buffer. For each radiolabeling, Na^125/131^I solution was dissolved 10–20 times with 0.001 M sodium carbonate-bicarbonate buffer with pH ∼9.0.

### Synthesis of 30 nm AuNPs, 2 nm PtNPs and 30 nm Au@Pt NPs

AuNPs, Au@Pt NPs and PtNPs were produced according to the procedure described in papers^26–29^ Briefly, AuNPs were synthesized in aqueous solution by the citrate reduction method. Briefly, 4.95 mg of HAuCl_4_·3H_2_O was alkalized with NaOH to pH ∼ 4.5 and heated for 30 min at 95 °C. Afterwards, sodium citrate dihydrate as a reductor was added (340 mM, 170 μL) and heating was continued for 3 hours. Au@Pt NPs were synthesized *via* reduction of Na_2_PtCl_6_·6H_2_O by ascorbic acid (AA). Initially, AuNP solution was heated (90 °C for 10 min) and Na_2_PtCl_6_·6H_2_O (0.32 mg, 1 mM) and AA (28 mg, 4 mM) were step-wise added at 10 and 30 min periods. Subsequently, the reaction mixture was heated for 30 min at 90 °C. PtNPs were synthesized by reduction of H_2_PtCl_6_ (19.5 mg, 5 mM) with C_6_Na_2_O_6_ (83 mg, 43.5 mM) at 90 °C for 60 min.

### Chemisorption of iodine radionuclides and stability studies

During each synthesis, the following amounts of nanoparticles (synthesized and characterized as we previously described^25^) were applied: 4.5 × 10^10^ (AuNPs), 1.9 × 10^10^ (Au@Pt), and 3.95 × 10^14^ (PtNPs) to have a similar outer surface area in every sample. First, for preconcentration and purification, nanoparticles were centrifuged and redispersed in deionized water. For 30 nm gold and core–shell nanoparticles, centrifuging for 10 min and 10 000 rpm was implemented, while for platinum nanoparticles, centrifugal concentrators Vivaspin®500 (cut-off MW = 3 kDa) were used for 20 min and 13 400 rpm. Subsequently, the radionuclide was added and the reaction was kept at room temperature for 1 hour with continuous mixing. Finally, HS-PEG-COOH (MW = 5 kDa) was added with the desired excess and the reaction was continued for the next 30 min.

Quality control was performed with the instant thin layer chromatography (iTLC) technique with the use of Storage Phosphor System Cyclone Plus (PerkinElmer, Waltham, MA, USA), glass microfiber chromatography paper impregnated with silica gel (iTLC SG, Agilent Technologies, Santa Clara, CA, USA), and methyl alcohol (MeOH) as the mobile phase. Analyses were performed after the first and final steps of radioconjugation as well as, during stability studies. For additional radiochemical yield evaluation, we centrifuged nanoparticles after each synthesis step and measured the activity of the collected fractions.

For stability studies radioconjugates were purified after synthesis and redispersed in 100 μL of deionized water. Next, 300 μL of human serum (HS) or phosphate-buffered saline (PBS) were added and the samples were heated at 37 °C for three days.

### Iodine release in a highly oxidative environment

Selected concentrations of hydrogen peroxide were prepared by dissolution of stock solution (30%) with deionized water. Then, radioconjugates after prior centrifugation, were dispersed directly in prepared H_2_O_2_ solutions. Samples were then incubated for 1 h at 37 °C. After this, quality control with iTLC was performed to find the released iodine ratio, as described above.

### *In vitro* toxicity

HepG2 and HeLa cells (8 × 10^3^) were seeded into 96-well plates and incubated overnight (37 °C, 5% CO_2_). Next, the growing medium was removed and radioconjugates in 100 μL of fresh medium were added. After 24, 48, and 72 h incubation, the medium was removed and a fresh one was added. Finally, 20 μL of CellTiter96® AQueous One Solution Reagent (Promega, MDN, USA) was added and absorbance was measured at 490 nm to calculate the % of metabolically active cells.

### DNA double-strand breaks

HepG2 cells (2.5 × 10^5^ per well) were seeded into six-well plates with sterile glass coverslips (diameter 12 mm), Thermo Fischer Scientific (Waltham, MA, USA) and incubated overnight. After removing the medium, cells were treated with radioconjugates (0–100 MBq mL^−1^), iodine-125 (100 MBq mL^−1^), and staurosporine (0.5 μM) and incubated for 12 and 24 h. Furthermore, the protocol was analogous to that reported previously.^30^ For γH2A.X foci detection with primary anti-phospho-Histone H2A.X (Ser139) antibody, clone JBW301 was dissolved 1 : 100 with blocking buffer (BB – 4% BSA in TBS) and 350 μL per well was added for overnight incubation at 4 °C. The next day, primary antibody was replaced with anti-mouse IgG secondary antibody conjugated with CF™ 633. Antibody was dissolved in blocking buffer according to manufacturer’s protocol and the cells were incubated for 2 h at room temperature with mixing. Finally, the cells were washed 3 times with water and for nuclei staining, Hoechst 33 258 was used. Imaging was performed on an FV-1000 confocal microscope (Olympus Corporation, Tokyo, Japan) with ex/em maxima: 630/650 nm for CF633 and ex/em maxima: 352/454 nm for Hoechst 33 258. The results were analyzed with Fiji 2.9.0 version.

### ^125^I–PtNP effects on a 3D tumor spheroid model

HepG2 cells (1 × 10^3^) suspended in 200 μL of growing medium were seeded into 96-well U-bottom ultra-low adherent plates (Corning®, Corning, NY, USA) seven days before the experiment, as reported previously.^31^ During incubation, 100 μL of medium was replaced every two days. After 7 days, radioconjugates in 100 μL growing medium were added. Spheroid images with area measurements were obtained and analyzed with a ZEISS Axio Vert.A1 Microscope and ZEN 2.1 software (Zeiss, Jena, Germany).

### Statistical analysis

Statistical analysis, one-way ANOVA and *t*-Student tests were performed with GraphPad Prism v.8 Software (GraphPad Software, San Diego, CA, USA). The results are presented as mean ± SD. *p* values are presented as: (*) *p* ≤ 0.05, (**) *p* ≤ 0.01, (***) *p* ≤ 0.001, and (****) *p* ≤ 0.0001.

## Results and discussion

### Chemisorption of ^131^I on the nanoparticle surface

Au and Pt are noble metals that exhibit similar atomic properties, such as covalent radius and electronegativity, which determine the type and strength of chemical bonds. A common feature of these metals is the formation of strong bonds with heavy halogens such as iodine and astatine (At).^32–34^ The Pt–I bond length (261 pm) is slightly shorter than that of Au–I (277 pm), which suggests the formation of a stronger Pt–I bond than Au–I. Analogously to Au, the chemisorption reaction of I^−^ onto the surface of Au@Pt NPs/PtNPs should be as follows (eqn (1)):

1 I^−^ + Pt^0^ + H_2_O → PtI + ½H_2_ + OH^−^

In our work, we utilized the high affinity of Pt atoms for iodine atoms to immobilize ^125^I on the surface of the NPs. Due to wider availability and cost-effectiveness, some of the chemical studies were performed on the ^131^I radionuclide having the same chemical properties. The studies were performed on 2 nm PtNPs, Pt-coated 30 nm AuNPs (Au@Pt NPs) and 30 nm AuNPs for comparison. Post synthesis, ^131^I was successfully immobilized on the NP surface with greater than 85% yield of chemisorption. Additionally, negligible changes occurred after subsequent surface functionalization with polyethylene glycol (PEG; PEG-ylation) for NP stabilization, dispersity enhancement, and purification realized in the final step (Table 1).

**Table 1:** Adsorption of iodine-131 on nanoparticles and the PEGylated nanoparticle surface^a^

	Adsorption [%]	
	Nanoparticles	PEGylated nanoparticles
AuNPs	93.46 ± 4.90	88.45 ± 0.69
Au@Pt	86.17 ± 4.75	85.26 ± 0.14
PtNPs	85.0 ± 4.3	81.82 ± 1.97

a Number of nanoparticles used in this study was calculated to reach the same outer surface area. The data are presented as mean ± SD of at least 3 different experiments.

As calculated, the average outer surface of a single NP accessible to iodine atoms was 12.5 nm^2^ for a 2 nm PtNP and 2.8 × 10^3^ nm^2^ for a 30 nm Au@Pt NP. Considering the theoretical cross-sectional area of a single I atom, ∼18 I atoms could be deposited per 1 nm^2^ of these NP surfaces. In our procedure, ∼0.9 iodine atoms per 1 nm^2^ were used to avoid oversaturating the surface.

Interestingly, both Pt-containing NPs (Au@Pt NPs and PtNPs) exhibited decreased chemisorption yields when compared to AuNPs used as a control in a parallel experiment. This phenomenon can be explained by the different types of stabilizing agents directly conjugated to the NP surface. Each type of NP is stabilized using a diverse agent, such as sodium citrate (*e.g.*, AuNPs), ascorbic acid (*e.g.*, Au@Pt NPs), and sodium rhodizonate (*e.g.*, PtNPs). Iodine chemisorption may occur after I^−^ oxidizes to I^0^ (from Na^131^I_aq_) and the subsequent direct deposition on the metal surface (eqn (1)), either *via* the replacement of stabilizing molecules^35^ or a combination both of the mentioned alternatives. Therefore, it is reasonable to consider the type of stabilizing agent as an indirect cause of the diversified yield for the different types of NPs investigated. After the PEG-ylation process, the release of ^131^I by the competitive displacement of iodine atoms by the thiol group did not occur. Inconsistencies were limited to the statistical differences in each of the described radioconjugates.

### Surface saturation

**Fig. 2:**
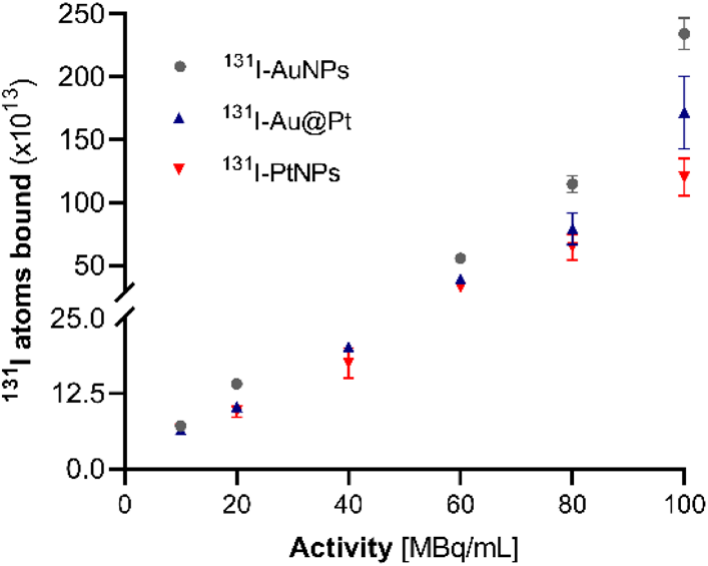
Surface saturation of AuNPs, Au@Pt I PtNPs with 10–100 MBq mL^−1 131^I (*n* = 3).

Assessing surface saturation was essential regarding the future application of ^125^I-NP radioconjugates for therapeutic procedures. Since very high radionuclide activities are necessary for effective Auger electron therapy, a high specific activity is required to further consider ^125^I-NP as a precursor for radiopharmaceuticals.^36^ Incubation of NPs with increasing activity of ^131^I indicated the formation of bound-to-free ^131^I equilibrium in solution regardless of the concentration (Fig. 2).

Sorption on the AuNP surface was the most efficient (>90%) for various concentrations, up to 100 MBq of ^131^I per 1 mL of NP solution. Au@Pt NPs and PtNPs exhibited inferior chemisorption efficiency. However, as observed, this is not related to the saturation and subsequent depletion of the NP surface available for the iodine. We observed the formation of an equilibrium state at each of the verified activities (20–100 MBq mL^−1^). Notably, the concentration of NP-bound I increased when the activity of ^131^I was greater while the yield remained at a comparable level. This result could partially be due to the aftermath of sideways reactions between the radionuclide and stabilizing agent, especially in the case of Au@Pt NPs and ascorbic acid, as reported previously.^37^

### Stability studies

**Fig. 3:**
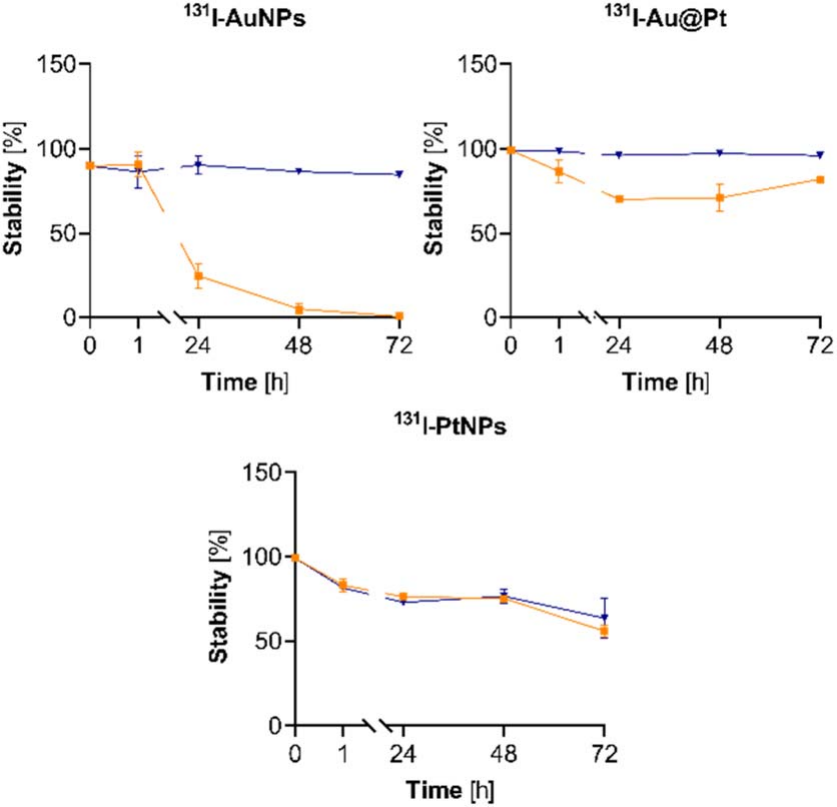
Stability studies of the synthesized radioconjugates in PBS and human serum (*n* = 3).

The synthesized radioconjugates demonstrated different stabilities in phosphate-buffered saline (PBS) and human serum (HS) (Fig. 3). Almost complete iodine desorption (>90%) from PEG-ylated AuNPs occurred after a longer than 1 h stability test. Pt-containing radioconjugates (Au@Pt NPs and PtNPs) revealed improved stability in HS when compared to AuNPs and reached ∼70% after 48 h. PBS did not induce a loss of stability in ^131^I–AuNPs and ^131^I–Au@Pt NPs, whereas ^131^I–PtNP stability remained at a constant level (75%) from 24 to 72 h. A lack of stability (^131^I–AuNPs) or minorly reduced stability (*e.g.*, ^131^I–Au@Pt and ^131^I–PtNPs) in HS can be related to the sulfur-containing peptides and proteins present in the fluid which can displace iodine from the NP surface. Like previously reported studies, ^131^I–AuNPs exhibited decreasing stability over time in fetal bovine serum (FBS).^38^ The stability of iodinated Pt-containing NPs has not been previously studied. The much greater stability of iodinated PtNPs and Au@Pt NPs in HS confirms the prediction that the Pt–I bond is stronger than Au–I. All of the tested PEG-ylated and iodinated NPs did not agglomerate in both PBS and HS during long time point experiments. This is in line with the results of previous publications concerned with AuNPs.^39,40^

### Iodine release in a highly oxidative environment

**Fig. 4:**
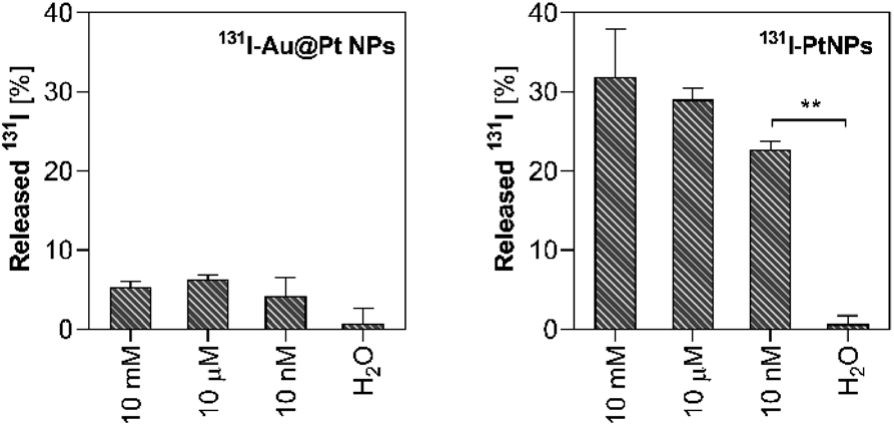
Iodine release under oxidative conditions induced with 10 mM–10 nM H_2_O_2_ from Au@Pt NP (left) and PtNP (right) radioconjugates (*n* = 3).

Due to their very short tissue range, AEs should be delivered into the cell nucleus, preferably close to the DNA.^41^ As previously reported,^25,42^ 2 nm NPs can penetrate the nuclear envelope of HepG2 cells making them very promising candidates for Auger electron therapy, especially when combined therapy can be considered. Successful delivery of iodine radionuclides closely or into the nucleus is also potentially achievable *via* iodine desorption in the cytosol of cancer cells with high oxidative status. Iodine reduction by H_2_O_2_ is widely described in the literature.^43,44^*In vitro* this process leads to the direct formation of HOI^45^ and other species *via* unstable intermediates.^46^ For this purpose, we decided to study whether ^125,131^I can be efficiently released from the NP surface under highly oxidative conditions corresponding to hepatic cancer cells (Fig. 4). Our previous studies concerned with designing novel HCC therapeutic approaches revealed that HepG2 cells have abnormally high oxidative status and significantly reduced antioxidant protection capacity.^25^

The physiological concentration of H_2_O_2_ in various cells is in the nanomolar range;^47^ thus 10 nM was considered a benchmark in this experiment. In order to verify whether iodine can be released in a dose-dependent manner in the presence of H_2_O_2_, we also implemented a higher concentrations range. Since we noticed that ^125^I–AuNPs is not a suitable model for *in vitro*-related studies due to its poor stability in biological media (Fig. 3), we avoided applying this radioconjugate in our further studies as a reference compound.

In each of the investigated H_2_O_2_ concentrations, iodine was effectively desorbed from PtNP radioconjugates reaching around ∼20% at 10 nM. The percentage of desorbed radionuclides increased with an increase in the H_2_O_2_ concentration, highlighting the impact of the oxidizing agent in this process. Contrary to PtNPs, a negligible release was observed for Au@Pt NPs (∼5%), and any desorption was found to occur when radioconjugates were incubated in water. The significant difference in iodine release between Au@Pt NPs and 2 nm PtNPs may refer to the partial dissolution of 2 nm PtNP in HepG2 cells postulated by Shoshan *et al.*^42^ Another possible explanation for the observed differences is related to the diverse physicochemical properties of both types of nanoparticles. 2 nm PtNPs due to a higher SA : V ratio should be more reactive against H_2_O_2_ as a result of their superior surface atom number compared to Au@Pt NPs. Both of the mentioned reasons (dissolution or reactivity) directly clarify iodine release occurring only in the case of 2 nm nanoparticles. If these circumstances occur after internalization, then a noteworthy improvement in therapeutic efficacy could be achieved due to the significant nuclear access for ^125^I.

Considering the discussed treatment strategy with ^125^I its major advantage in relation to 5-iodo-2′-deoxyuridine labelled with ^125^I (^125^IUdR) must be emphasized. This most frequently occurring way to implement the therapy with ^125^I without a targeting moiety has numerous side effects related mostly to systemic toxicity as a consequence of poor selectivity. Keeping in mind the necessity of overcoming this limitation, our strategy can be greatly implemented in future therapeutic approaches, as it can potentially ensure and improve cancer treatment selectivity.

### *In vitro* toxicity

Investigation of radioconjugate-mediated cell viability effects was performed using HepG2 cells, while adenocarcinoma cells (HeLa) which do not exhibit oxidative properties were implemented as a control in this experiment. As we previously reported, oxidative status plays a key role in Pt-containing NP induced toxicity. Previously we observed that high oxidative potential is required for triggering Au@Pt NP/PtNP biological activity, as only HepG2 cells were affected by the treatment with various concentrations of NPs; cells with increased but moderate oxidative status cells (SKOV-3) remained unaffected.^25^ Iodine release from PtNPs occurring only under highly oxidative conditions was another criterion taken into account in the selection of the *in vitro* model. Thus, following the strategy of targeting the tumor microenvironment, we verified the therapeutic potential of the synthesized radioconjugates against hepatic cancer cells (HepG2). HeLa cells, notwithstanding being a cancer cell line, are one cell line that is very similar to healthy cells.^48^ Hence, we also evaluated the synthesized radioconjugates with HeLa cells as a reference cell line.

**Fig. 5:**
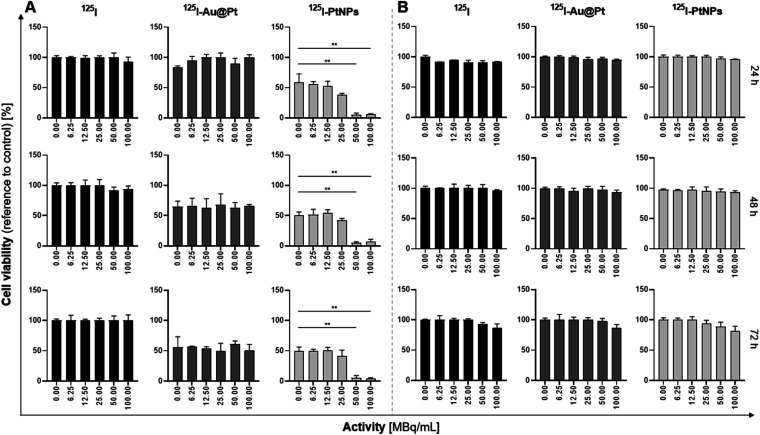
Viability studies of HepG2 (A) and HeLa (B) cells treated with 0–100 MBq mL^−1^ of ^125^I and radioconjugates – ^125^I–Au@Pt and ^125^I–PtNPs. Data presented for “0” MBq mL^−1^ correspond to non-radioactive compounds used at the same Pt concentration (145 μg mL^−1^) as that during radioconjugate evaluation (*n* = 3).

HepG2 cell viability (Fig. 5A) was not affected after treatment with free ^125^I^−^ at any of the tested radioactivity concentrations (6.25–100 MBq mL^−1^). We also did not find any radioactivity-related response during the incubation with ^125^I^−^ Au@Pt NPs. The decrease in the mitochondrial activity fraction over time was related to the chemotoxicity of Au@Pt NPs with any enhancement of the effect achieved by electrons emitted from ^125^I decay. This is a consequence of ^125^I location outside of the nucleus, due to the intranuclear absence of Au@Pt NPs^25^ and lack of I release (Fig. 4). Because of the characteristics of ^125^I decay, therapeutic effectiveness was probably impossible to achieve at sites distant from the DNA. Decay of ^125^I results in the emission of ∼23 AEs with an average energy in the range 0.07–30.1 keV and around 7.3 IC electrons with 35 keV maximum energy.^49,50^ Comparing this with other Auger electron emitters, it was found that the low energies of the emitted particles make them unfavorable for expecting a high biological response induced by nuclides located away from DNA.^51^

A significant decrease in the mitochondrial redox activity was observed at 50 MBq mL^−1^ and 100 MBq mL^−1^ after 24 h of incubation with 2 nm NPs (*p* ≤ 0.01). Bare PtNPs (0 MBq mL^−1^) induced a ∼50% decrease in cell viability while ^125^I–PtNPs improved the therapeutic effect to ∼90%. It has also been observed that 25 MBq mL^−1^ exhibits a slightly additive effect, but contrary to 50 and 100 MBq mL^−1^, the effect was much weaker with a ∼10% improvement in the cell viability decrease when compared to PtNPs without radioactive I (*p* > 0.05). These effects were triggered by prooxidative activity of bare PtNPs and radiotoxicity of ^125^I. Auger and IC electrons could induce significant treatment enhancement through the intranuclear uptake of PtNPs of which ∼13–15% are located in the HepG2 cell nucleus after 24 h^25^ or due to iodine release from the nanoparticle surface. Core–shell nanoparticles’ presence in the nuclei fraction was not detected reaching only ∼2% and oscillating within the error limit. These data directly demonstrate that AEs are most effective when localized in direct proximity to DNA.^52^ It is applicable, especially when weaker AEs are considered.^53^

HeLa cells were not affected by Au@Pt NPs or PtNPs (Fig. 5B). Application of 0–100 MBq mL^−1 125^I did not decrease the mitochondrial activity of HeLa cells. In contrast to HepG2 cells, the low concentration of H_2_O_2_ and resulting from other anatomical functions of primary tissue a different oxidative potential in HeLa cells did not cause the platinum biological activity and release of ^125^I with subsequent penetration into the nucleus or DNA. The presented findings support the hypothesis that the designed system is highly specific since its biological activity is triggered only under favorable conditions in cytosol.

Moreover, in agreement with our predictions, short-range AEs, even considering high LET and high activity doses, are unable to kill the cells without the precise targeting DNA achieved using a targeting vector or cell sensitivity expressed under specific physiological conditions. Cytotoxicity studies revealed that sorption of iodine does not impair biological activity of PtNPs, maintaining their pro-oxidant capacity. The additive effect observed in HCC cells from oxidative stress induced with PtNPs and radiotoxicity from ^125^I gives an exciting opportunity to implement this strategy as combined therapy. Translation of these results into *in vivo* and preclinical evaluation needs to answer the question about the safety tolerance limit of nanoparticle concentrations. The range (145 μg mL^−1^) used in our current and previously reported studies is relatively low when compared to the data published by other groups.^54,55^ Beneficially, our concept to implement ^125^I–PtNPs gives an excellent opportunity to obtain high specific activities required during the further development of this strategy in order to achieve satisfactory parameters for nuclear medicine.

### Morphological changes

**Fig. 6:**
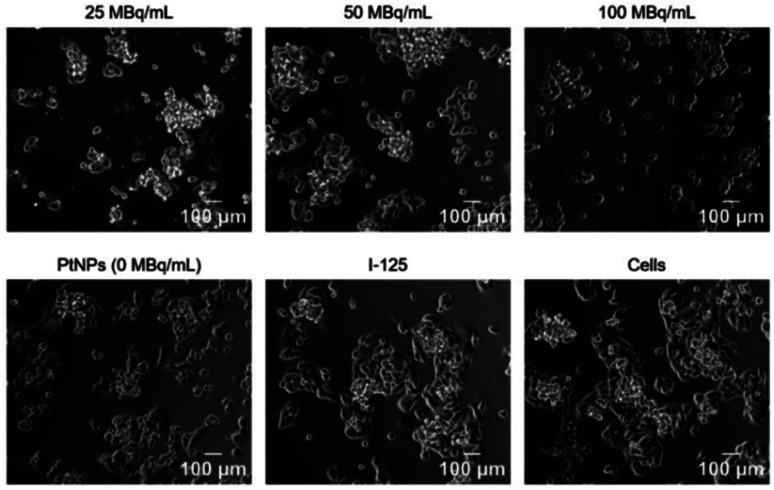
Morphological changes in HepG2 cells induced by non-radioactive (0 MBq mL^−1^) PtNPs as a “chemotoxic effect” and radioconjugates (25–100 MBq mL^−1^) as presentation of a dose-dependent manner of the induced chemo-Auger electron effect.

Treatment with radioconjugates changed not only cell mitochondrial activity but also affected alterations in cellular morphology. Microscopic images of cells incubated for 24 h with various concentrations of ^125^I–PtNPs revealed substantial morphological changes in HepG2 cells when compared to non-radioactive PtNPs, ^125^I (100 MBq mL^−1^), and untreated cells (Fig. 6). Increasing concentrations of ^125^I-radioconjugates have resulted in intensifying modifications of cellular morphology. HepG2 is a well-known cell line that typically grows by forming clusters and layers of cells. Treatment with radioconjugates disrupted (25 MBq mL^−1^) or destroyed (50 and 100 MBq mL^−1^) the integrity of these clusters. Increasing concentrations also prompted cell membrane shrinkage and a distinctive reduction in the cell number. Small changes were also observed in the control cells incubated with non-radioactive PtNPs, consisting of a minor reduction in the size of clusters followed by the separation of single cells outside from groups. This was a consequence of the chemotoxicity of PtNPs achieved by oxidative stress and intranuclear location, which was previously reported.^25^ As expected, no modifications were found in the cells treated with 100 MBq mL^−1^ of ^125^I according to the MTS assay (Fig. 6). The low range of AE and IC electrons makes Auger therapy poorly or not effective without nuclear location, even for high LET particles, such as Auger electrons.

### DNA double-strand breaks (DNA DSBs)

**Fig. 7:**
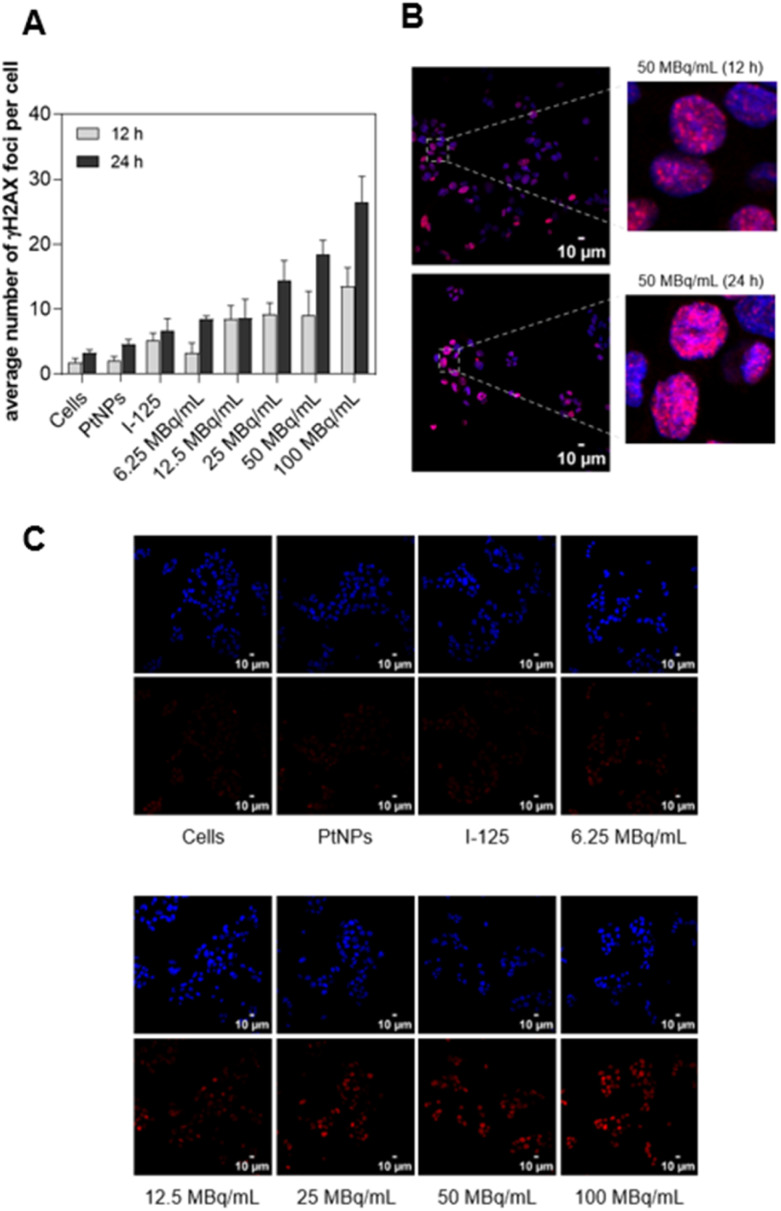
DNA DSBs visualized with γH2A.X staining – the average number of γH2A.X foci per cell at different times and activity concentrations (A); merged images of γH2A.X foci distribution over the genetic material of treated cells (B and C) (*n* = 3).

Despite progressive dysfunction of the mitochondria followed by significant morphological changes, induction of DNA DSBs is considered one of the most desired outcomes of radiopharmaceutical anticancer activity.^56^ Lethal and unrepairable damage of the genetic material is the most effective way to achieve a high therapeutic response. A growing number of DSBs in HepG2 cells was found with an increase in the radioactivity concentration, mostly in the range of 12.5–100 MBq mL^−1^ (Fig. 7A).

The average counts of phosphorylated H2A.X Histone in a single nucleus were 14 and 26 at 100 MBq mL^−1^ after 12 and 24 h, respectively. We also observed up to 5 spontaneous DSBs in untreated cells. After 12 h of treatment, only a slight increase in γH2A.X foci was observed for most of the analyzed radioconjugate concentrations, when compared to ^125^I (100 MBq mL^−1^). Longer incubations (24 h) revealed an increasing signal of DNA damage in a dose-dependent manner, primarily for 25 MBq mL^−1^, 50 MBq mL^−1^, and 100 MBq mL^−1^ (Fig. 7A and B) which corresponds to the data obtained during cytotoxicity investigation. Considering the redox activity of PtNPs, we could also expect an increased DSB ratio in cells treated with the non-radioactive compound. It is widely reported that ROS may induce serious DNA damage, including DNA DSBs.^57,58^ In our studies, the level of γH2A.X foci in the cells treated with PtNPs was similar to that in untreated control cells. This can be explained by the kinetics of oxidative stress induced by applied NPs. First, low ROS overproduction was observed after 12 h with a subsequent large increase from 24 to 72 h.^25^ Thus, we conclude that 24 h of incubation was too short of a treatment for efficient DNA DSB generation *via* free radicals produced by non-radioactive PtNPs in the cytosol. The radioconjugate uniformly affected the treated cells, and the red signal from γH2A.X was homogenously detected in most of the cells (Fig. 7B and C). This is a very valuable outcome, as it confirms that the observed desirable signals are not accidental and not locally situated. Our results agree with those of previously reported models and calculations related to the ability of AEs to induce DNA DSBs.^59,60^ However, the radionuclide-DNA distance plays a crucial role in effective DNA damage induction, which explains the lack of an effect from radioconjugates located outside of the nucleus, such as ^125^I and ^125^I–Au@Pt NPs.^61^

### ^125^I–PtNP effects on a 3D tumor spheroid model

Taking into account the low tissue range of AEs and the uncertain penetration of tumors by NPs, AEs, and IC electrons, we examined whether the synthesized radioconjugates would be effective against a 3D spheroid model. As we previously reported, Au@Pt NPs and PtNPs can effectively induce spheroid toxicity, but any decrease in the tumor area was related to high chemotoxicity revealed with fluorescence imaging.^25^ Since the expected therapeutic effect improvement in the case of ^125^I–Au@Pt NPs was not achieved, we focused our attention on ^125^I–PtNPs.

**Fig. 8:**
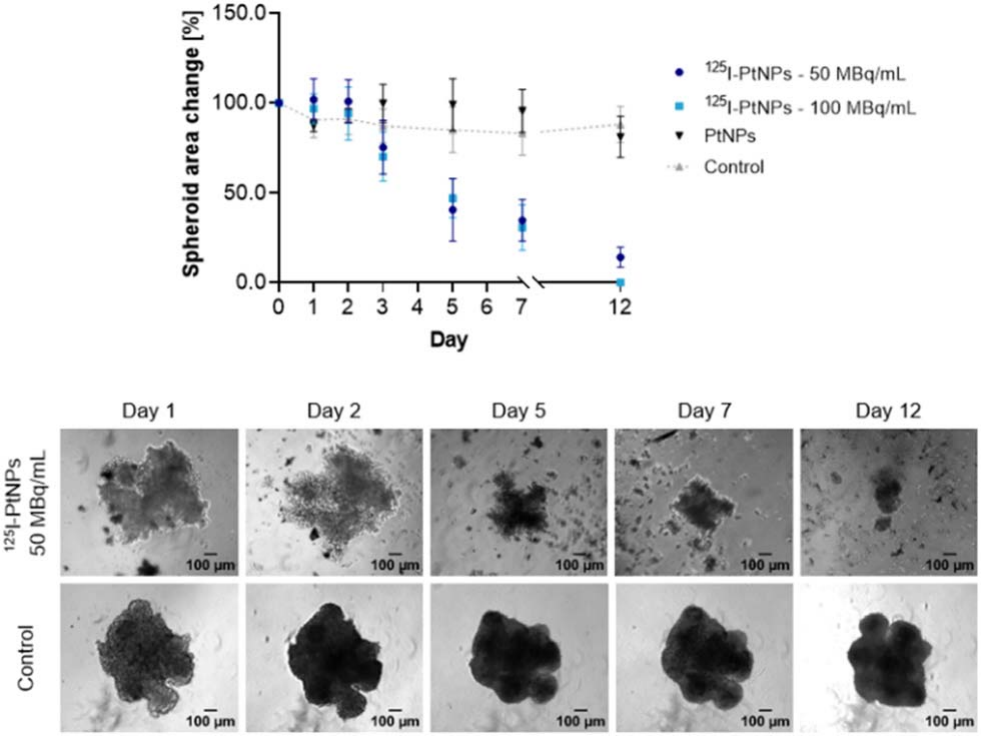
Effects of ^125^I–PtNPs radioconjugates against a HepG2 tumor spheroid model (*n* = 3).

Because of ^125^I characteristics and cytotoxicity studies (Fig. 5), we applied 50 and 100 MBq mL^−1^ of ^125^I–PtNPs for continuous treatment until significant tumor area reduction was distinguished. Fig. 8 describes spheroid area changes in treated and control samples. We observed that the first symptoms of tumor growth inhibition were induced 72 h post-NP injection. These changes progressed more slowly when compared to the MTS assay, which was reasonable and expected. The spheroid area was then reduced by ∼10 and 15% for 50 MBq mL^−1^ and 100 MBq mL^−1^, respectively, in reference to control cells. However, during this time, tumors maintained their initial structure without any morphological fluctuations. The first meaningful changes in the tumor shape and integrity were noted on the 5th day. In both of the tested concentrations, many cells detached from the tumor and ∼50% of the area decreased. Subsequently, 100 MBq mL^−1^ of radioconjugate led to the total disintegration of the spheroid on the 12th day, whereas 50 MBq mL^−1^ reduced tumor size by approximately sevenfold.

## Conclusions

Due to the insidious growth nature of HCC, most patients are diagnosed at advanced stages of the disease, at which point the available therapeutic options are limited and ineffective.

Cutting-edge research and development in HCC nanomedicine has provided a powerful tool compared to traditional methods such as systemically applied sorafenib, transarterial chemo, and radioembolization. The combination chemo-Auger therapy showed additive chemotoxic effects of PtNPs and a radiotherapeutic action of Auger electrons emitted from ^125^I occurring only in an elevated oxidative environment of HCC. Release of ^125^I from the surface of PtNPs as a result of oxidation promotes better accessibility of the radionuclide to the structures critical for maintaining cell functions (*e.g.* the cell nucleus and DNA). Therefore, a selective effect of ^125^I–PtNPs on HepG2 liver cancer cells is observed.

Prospective application may be realized by the transarterial injection of ^125^I-NP lipiodol emulsions which have a suitable particle size for the embolization of the middle and small arteries of HCC. In addition, lipiodol has the advantage of selectively accumulating in HCC tissues, which enables ^125^I-NP lipiodol emulsions to be efficiently absorbed by tumor tissues. What we propose is to apply ^125^I–PtNPs as a drug loaded into micrometer range lipiodol emulsions allowing embolization with further chemo-Auger induced toxicity. Our results highlight the exciting prospects for the development of Pt NP-based therapeutics as highly effective, safe for healthy tissues and selective tools. Versality of the proposed radioconjugates gives an opportunity to personalize the treatment strategy through the selection of the administration route and may be associated with using SPECT properties of ^125^I to image not only the radioconjugate location, but also the following treatment progress.

## Author contributions

Conceptualization: K. W. and A. B.; formal analysis: K. W., K. Ż.-M. and M. W.; funding acquisition: A. B.; investigation: K. W., A. M.-P., and K. Ż.-M.; methodology: K. W.; project administration: A. B.; resources: A. B.; supervision: K. W. and A. B.; visualization: K. W.; writing—original draft: K. W.; writing—review and editing: K. W. and A. B. All authors have read and agreed to the published version of the manuscript.

## Conflicts of interest

There are no conflicts to declare.

## Supplementary Material
